# EPICE-HIV: An Epidemiologic Cost-Effectiveness Model for HIV Treatment

**DOI:** 10.1371/journal.pone.0149007

**Published:** 2016-02-12

**Authors:** Björn Vandewalle, Josep M. Llibre, Jean-Jacques Parienti, Andrew Ustianowski, Ricardo Camacho, Colette Smith, Alec Miners, Diana Ferreira, Jorge Félix

**Affiliations:** 1 Exigo Consultores, Lisbon, Portugal; 2 Fundació Lluita contra la SIDA, Hospital Universitari Germans Trias i Pujol, Universitat Autònoma de Barcelona, Barcelona, Spain; 3 Department of Clinical Research and Biostatistics, Côte de Nacre University Hospital, Caen, France; 4 Regional Infectious Disease Unit, North Manchester General Hospital, Manchester, United Kingdom; 5 Rega Institute for Medical Research, Katholieke Universiteit Leuven, Leuven, Belgium; 6 Department of Infection and Population Health, University College London, London, United Kingdom; 7 Department of Health Services Research and Policy, London School of Hygiene and Tropical Medicine, London, United Kingdom; University of Athens, Medical School, GREECE

## Abstract

The goal of this research was to establish a new and innovative framework for cost-effectiveness modeling of HIV-1 treatment, simultaneously considering both clinical and epidemiological outcomes. EPICE-HIV is a multi-paradigm model based on a within-host micro-simulation model for the disease progression of HIV-1 infected individuals and an agent-based sexual contact network (SCN) model for the transmission of HIV-1 infection. It includes HIV-1 viral dynamics, CD4^+^ T cell infection rates, and pharmacokinetics/pharmacodynamics modeling. Disease progression of HIV-1 infected individuals is driven by the interdependent changes in CD4^+^ T cell count, changes in plasma HIV-1 RNA, accumulation of resistance mutations and adherence to treatment. The two parts of the model are joined through a per-sexual-act and viral load dependent probability of disease transmission in HIV-discordant couples. Internal validity of the disease progression part of the model is assessed and external validity is demonstrated in comparison to the outcomes observed in the STaR randomized controlled clinical trial. We found that overall adherence to treatment and the resulting pattern of treatment interruptions are key drivers of HIV-1 treatment outcomes. Our model, though largely independent of efficacy data from RCT, was accurate in producing 96-week outcomes, qualitatively and quantitatively comparable to the ones observed in the STaR trial. We demonstrate that multi-paradigm micro-simulation modeling is a promising tool to generate evidence about optimal policy strategies in HIV-1 treatment, including treatment efficacy, HIV-1 transmission, and cost-effectiveness analysis.

## Introduction

As a result of the introduction of increasingly potent and safe anti-retroviral therapy (ART), over the last decade HIV-1 infection has largely become a manageable disease, with mortality rates approaching those of the general population in many countries [1–3]. Similar to other pharmaceutical drugs, before marketing authorization, efficacy and safety profiles of new anti-retroviral regimens are usually investigated exclusively in clinical trial settings.

Most current clinical trials on HIV-1 ART follow a predefined schedule of follow-up visits rarely spaced more than 2 to 3 months apart, with quite rapid confirmation of virologic failure and drug withdrawal after initial detection [4–7]. Common clinical trial efficacy endpoints and their algorithms (e.g. the percentage of virologically suppressed patients at a certain time window, determined by the FDA Snapshot algorithm) are crucially dependent on these pre-specified follow-up protocols [4, 5, 7, 8]. International HIV-1 treatment guidelines, however, suggest follow-up visits for plasma HIV-1 RNA viral load assessment every 3 to 6 months (with more frequent monitoring only at the start of ART) and confirmation of virologic failure 1 to 2 months later [9]. Moreover, in clinical practice, much heterogeneity tends to be observed with respect to the monitoring of ART, and subjects on stable and suppressive regimens are commonly seen every 6 months [10, 11].

Despite efforts to raise patient´s adherence [12–16], in clinical practice it continues to be lower in comparison to clinical trials [17, 18]. For many patients it may be quite difficult to maintain a high level of adherence, necessary to achieve durable viral suppression, over the life-time course of a chronic disease [19–23]. Suboptimal adherence not only limits the effectiveness of therapy in terms of viral suppression, but also facilitates the replication and selection of resistant mutant viral strains, often limiting subsequent ART options [24, 25]. HIV resistance drug resistance has further become a relevant public health issue, as HIV-1 resistance mutations can be transmitted to other individuals [26].

Due to the heterogeneity observed in real-life monitoring schedules and adherence patterns, basing cost-effectiveness models for HIV-1 treatment mainly on the efficacy observed in clinical trials has the potential of leading to biased estimates of true effectiveness. Moreover, for drugs whose public use has only recently been authorized, little to no data might be available from observational studies, further restricting modeling options.

State of the art cohort and micro-simulation cost-effectiveness models for HIV-1 treatment typically focus on clinical outcomes such as viral suppression, life expectancy, causes of death, opportunistic infections and time on treatment [27–31]. Many of these models lack the ability of quantifying the impact of different treatment regimens and adherence behavior on the transmission of HIV-1 and HIV-1 drug resistance [27–31].

Some models that include transition modules tend to do so in a mostly deterministic, non-dynamic nature, making use of systems of ordinary differential equations. These models are capable of distinguishing between different populations (uninfected, infected, heterosexual, homosexual, injection drug users), linking them through pathway-dependent infection rates, but lack the ability to capture important population behavioral heterogeneity [27, 29, 31].

In this paper, we present EPICE-HIV, an epidemiologic cost-effectiveness model for HIV-1 treatment, designed to produce qualitatively and quantitatively credible results without overly depending on outcomes of clinical trials and observational studies. EPICE-HIV is a multi-paradigm model based on a within-host micro-simulation for the disease progression of HIV-1 infected individuals and an agent-based sexual contact network (SCN) model for the transmission of HIV-1 infection (transmission module). The two parts of the model are joined through a per-sexual-act, plasma HIV-1 RNA viral load dependent probability of HIV-1 transmission in serodiscordant couples and a CD4^+^ T cell count dependent mortality rate.

Since the algorithm driving the HIV-1 transmission module has been described and validated previously in the context of sexually transmitted diseases [32], this paper will focus on the within-host model of EPICE-HIV, in which disease progression is driven by adherence to ART and the interdependent changes in CD4^+^ T cell counts and plasma HIV-1 RNA levels. For validation purposes, a simulation study was set up to investigate the effectiveness of once daily treatment with rilpivirine 25mg (RPV), emtricitabine 200mg (FTC) and tenofovir DF 245mg (TDF) as initial ART, mimicking the conditions and population of the STaR study [5, 33].

## Material and Methods

**Fig 1:**
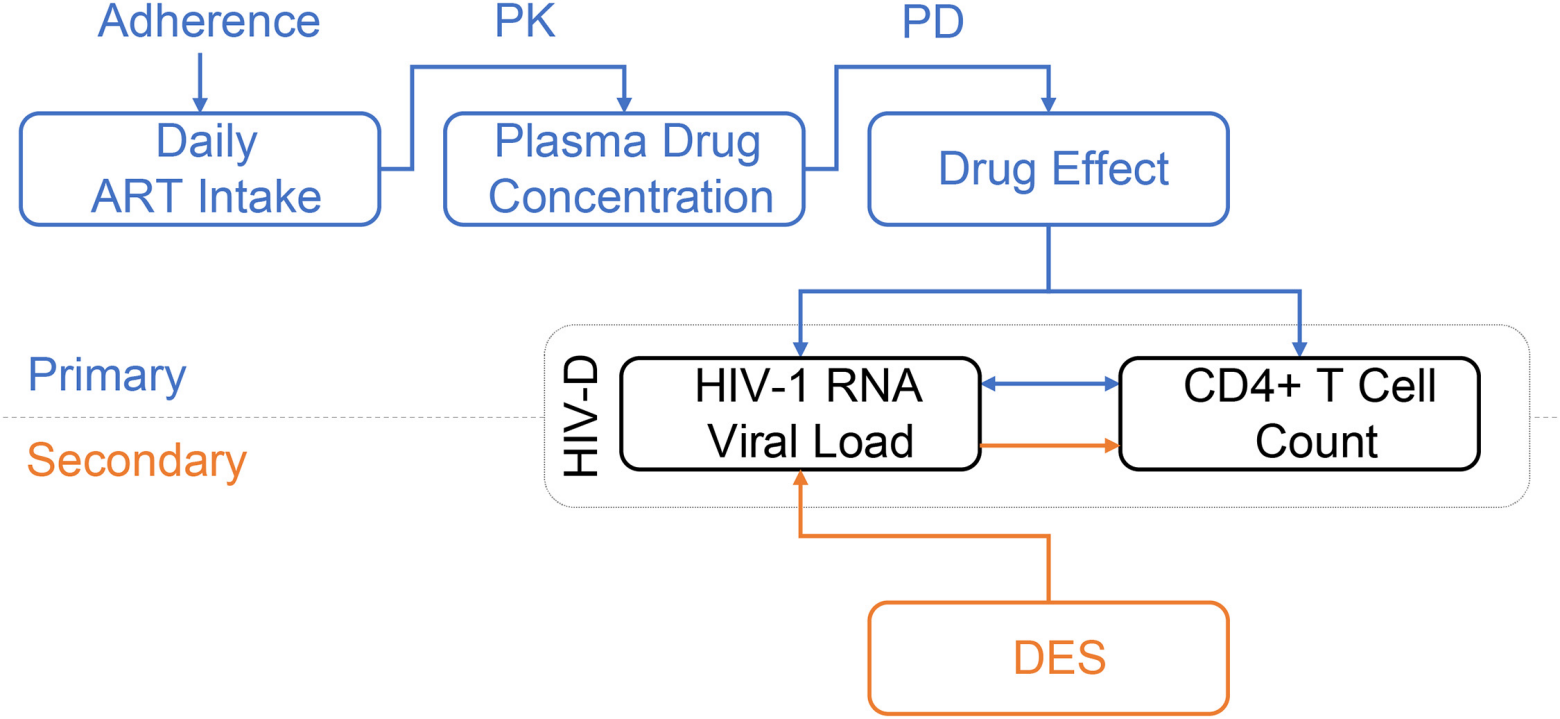
Diagram of the within-host model for disease progression of HIV-1 infected individuals. For the treatment regimen of primary interest (blue diagram), the adherence model dictates the daily intake of ART. Through a pharmacokinetic (PK) and pharmacodynamic (PD) model, the plasma drug concentration and drug effect are determined. Drug effect influences the inter-dependent and time-evolving plasma HIV-1 RNA level and CD4+ T cell count in the basic HIV-1 dynamics model (HIV-D). For treatment regimens of secondary interest (orange diagram), a discrete event simulation model (DES) determines the plasma HIV-1 RNA level over time. Using the dynamics of the HIV-D model, CD4+ T cell counts are updated accordingly.

In the within-host model (Fig 1), adherence to the ART regimen of primary interest is modeled using a binary autoregressive model, assuming adherence on any given day is conditional on adherence on the previous day. A per-drug, one-compartment, pharmacokinetics model (PK) followed by a pharmacodynamics model (PD), link adherence to a progressive HIV-1 dynamics model (HIV-D) generating time dependent plasma concentrations of free virus and uninfected and infected CD4^+^ T cells. Treatment regimens of secondary interest are modeled connecting a previously published discrete event simulation (DES) model for HIV-1 treatment [34] to the HIV-D model.

### Basic HIV-1 Dynamics Model

The HIV-D model consists of four populations: uninfected CD4^+^ T cells (*T*_1_), productively infected activated CD4^+^ T cells (*T*_2_), latently infected resting CD4^+^ T cells (*L*) and HIV-1 virions (*V*) [35]. Initially, only uninfected target CD4^+^ T cells (*T*_1_) and HIV-1 virions (*V*) are present in the system (Table 1).

**Table 1:** Basic HIV-D model populations and initial values [35].

Population	Description	Initial value
***T***_**1**_	Uninfected CD4+ T cells	1,000 cells/mm^3^
***T***_**2**_	Productively infected CD4+ T cells	0 cells/mm^3^
***L***	Latently infected CD4+ T cells	0 cells/mm^3^
***V***	HIV-1 virions^‡^	0.001 virions/mm^3^

^‡^ Corresponding to 2 plasma HIV-1 RNA copies/mL.

HIV-1 virions can infect target CD4^+^ T cells, a process modeled through a commonly used “mass-action” term $k_{T_{1}}VT_{1}$, with $k_{T_{1}}$ the target CD4^+^ T cell infection-rate by free virus. Upon infection, a small fraction *α*_*L*_ of CD4^+^ T cells will result in latently infected CD4^+^ T cells (*L*), not actively producing HIV-1 virions. The remaining fraction of infections results in productively infected CD4^+^ T cells (*T*_2_). Latently infected CD4^+^ T cells are activated into productively infected cells at a constant rate *α*_*L*_.

All cells have a finite lifespan, determined by death-rates $\delta_{T_{1}},\;\delta_{T_{2}}$ and *δ*_*L*_ for uninfected, productively infected and latently infected CD4^+^ T cells, respectively. For HIV-1 virions, the death-rate is given by *δ*_*V*_. Source rates of uninfected CD4^+^ T cells ($f_{s}s_{T_{1}}$) and HIV-1 virions (*f*_*p*_*p*_*v*_*T*_2_), drug potency (*ε*) and persistent low level viremia (*V*_*Min*_) are discussed further on in this section.

**Fig 2:**
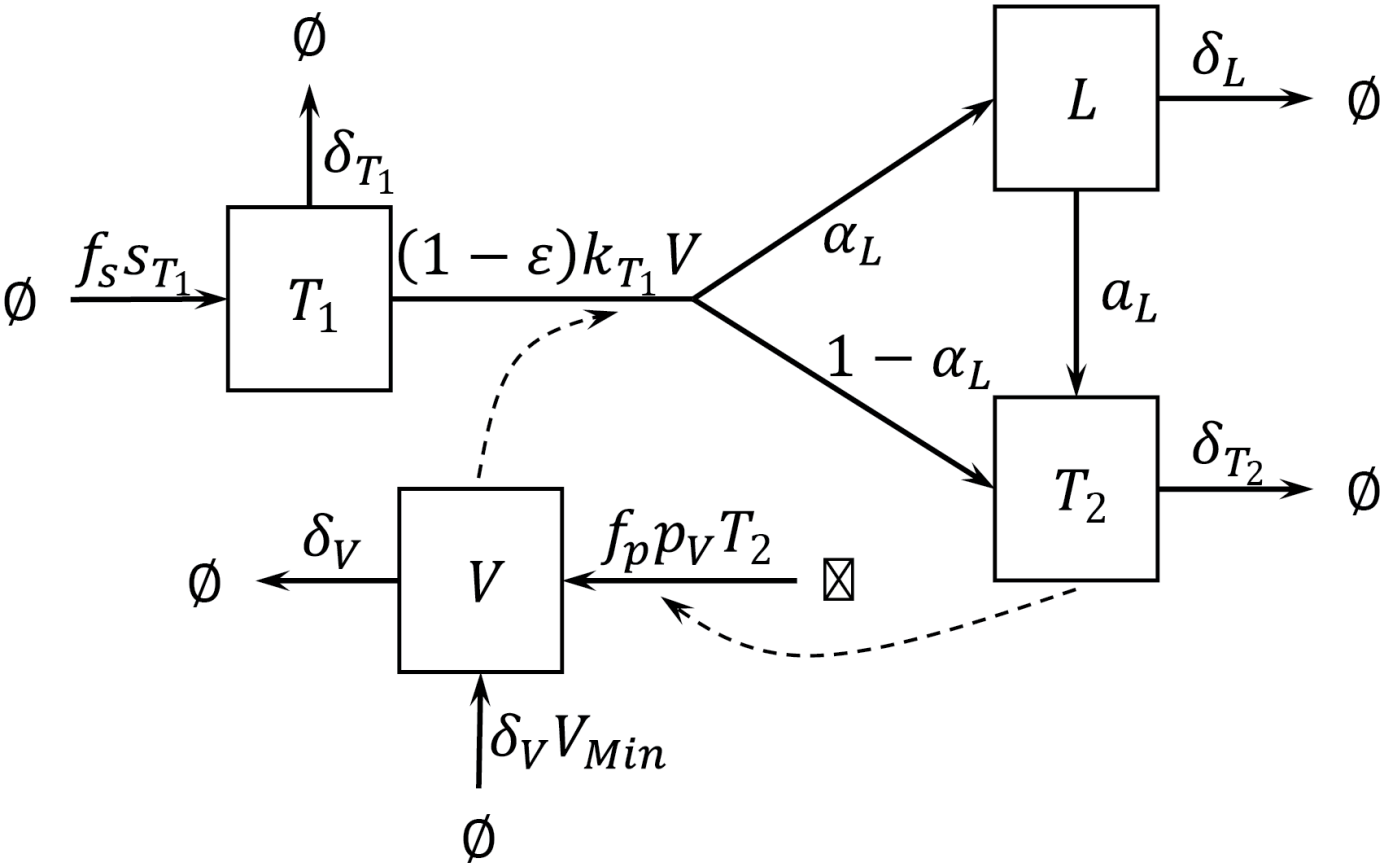
Stock and flow diagram of the basic HIV-1 dynamics model, modified from Rong et al [35]. Uninfected CD4+ T cells (T_1_) are produced at a rate $f_{s}s_{T_{1}}$. Infected CD4+ T cells are produced at a rate $k_{T_{1}}VT_{1}$, following mass-action kinetics between free virions (V) and uninfected CD4+ T cells. A small fraction α_L_ of infections results in latently infected resting CD4+ T cells (L). The remaining fraction results in productively infected activated CD4+ T cells (T_2_). Latently infected CD4+ T cells are activated into productively infected cells at a constant rate a_L_. Free virions are produced by productively infected CD4+ T cells at a rate f_p_p_v_T_2_.

The following system of ordinary differential equations, of which a graphical illustration is given in Fig 2, represents the resulting rate of change in the concentration of cells for each of the four modelled populations:

$$\begin{array}{lll} \frac{dT_{1}}{dt} & = & f_{s}s_{T_{1}}-\left(1-\varepsilon \right)k_{T_{1}}VT_{1}-\delta_{T_{1}}T_{1}\\ \frac{dT_{2}}{dt} & = & \left(1-\alpha_{L}\right)\left(1-\varepsilon \right)k_{T_{1}}VT_{1}-\delta_{T_{2}}T_{2}+a_{L}L\\ \frac{dL}{dt} & = & \alpha_{L}\left(1-\varepsilon \right)k_{T_{1}}VT_{1}-\delta_{L}L-a_{L}L\\ \frac{dV}{dt} & = & f_{p}p_{V}T_{2}-\delta_{V}V+\delta_{V}V_{Min}. \end{array}$$ (1)

#### Disease Progression

During the first stage of HIV-1 infection (acute infection), in the absence of treatment, large amounts of virus are being produced, destroying CD4^+^ T cells in the process. As a result, CD4^+^ T cell counts drop rapidly and viral load levels spike. At the end of this stage CD4^+^ T cell counts begin to increase, but do not return to pre-infection levels. At the same time, plasma viral loads drop back down and settle into a viral set-point after a couple of months, remaining at more stable levels thereafter. HIV-1 infection then moves into a clinical latency stage, in which HIV-1 continues reproducing, but at much lower levels than during the acute stage. This stage, though very variable, typically lasts about 8–10 years and is characterized by a slow depletion of CD4^+^ T cells and late stage increase in plasma viral load [36–38].

Over time the efficiency in viral production increases due to different factors, including the progressive loss by the immune system of the ability to contain viral production. In the model, this is implemented by applying a time-dependent replication increase factor (*f*_*p*_) to the initial viral production rate (*p*_*v*_) of productively infected CD4^+^ T cells (*T*_2_) [39]. Furthermore, since HIV-1 infection is known to eventually exhaust the body’s capacity to produce new uninfected CD4^+^ T cells, a time-dependent capacity reduction factor (*f*_*s*_) is also applied to the base CD4^+^ T cell source rate ($s_{T_{1}}$) [39].

Both adjustment factors (*f*_*p*_ and *f*_*s*_) depend on a measure for the total viral burden accumulated over time since the start of infection, as

$$\begin{array}{lll} f_{s} & = & {\left(1+Q\right)}^{-1}\\ f_{p} & = & 1+pQ \end{array}$$ (2)

where *Q* represents a Weibull cumulative distribution function on the viral burden *V*_*b*_, the area under the curve of the time evolving virion concentration *V* with respect to reference value *V*_*ref*_ [39]:

$$\begin{array}{ccc} Q\left(V_{b}\right) & = & 1-\text{exp}\left[-{\left(\frac{V_{b}}{\sigma }\right)}^{k}\right]\\ V_{b}\left(t\right) & = & \frac{1}{V_{ref}}\int_{t}^{0}V\left(x\right)dx. \end{array}$$ (3)

The effect of specific incumbent period determinants that may affect disease progression (e.g. age at infection, the presence of other sexually transmitted diseases) have not been included in the current paper and will be addressed in future work.

#### Drug Potency

Anti-retroviral drugs within different classes (nucleoside reverse transcriptase inhibitors (NRTI), non-nucleoside reverse transcriptase inhibitors (NNRTI), protease inhibitors (PI), etc.) act on different points in the complex viral replication process in CD4^+^ T cells. In order not to overly complicate the HIV-D model, an overall effect of ART on plasma CD4^+^ T cell and HIV-1 virion concentrations was assumed. This overall drug effect or drug potency *ε* represents the percentage of new cell infections inhibited and acts upon the mass-action term $k_{T_{1}}VT_{1}$ by reducing the rate $k_{T_{1}}$ at which uninfected target CD4^+^ T cells are infected by free virus [35].

The analysis of viral decay following initiation of ART shows that plasma HIV-1 RNA levels typically decline in three distinct phases [35]. In the model, to simulate the second phase decay, a population of latently infected CD4^+^ T cells was considered, with a much lower decay rate than the productively infected CD4^+^ T cells.

#### Persistent Low Level Viremia

Studies with especially sensitive assays [40] have demonstrated that the second phase decay typically brings plasma viral loads down to a new quasi-steady state below the 50 plasma HIV-1 RNA copies/mL threshold, with an average of persistent low level viremia of around 3 copies/mL [41]. This could reflect the release of viruses from other stable reservoirs, potentially outside of the plasma, which are unaffected by treatment intensification. To accomplish a persistent low level of viremia in the model, an extra term *δ*_*V*_*V*_*min*_ was added to the rate of change in concentration of virions, representing the potential inflow of free viruses from external reservoirs.

The level of persistent low viremia correlates well with pre-therapy plasma HIV-1 RNA levels, more than with specific treatment regimens, even though some reports have linked PI with higher rates of low level viremia than NNRTI [42]. Moreover, longitudinal analysis revealed no significant decline in the level of persistent viremia over time (60–110 weeks), while on suppressive ART. As a result of this, the HIV-D model assumes that the level of persistent low-viremia under ART (represented by the parameter *V*_*min*_) remains constant over time and depends only on pre-therapy plasma HIV-1 RNA levels, through a log-normal model fitted to data presented in Maldarelli et al. (2007) [41]. The results of this analysis are presented in Table 2 and show a highly significant effect of pre-therapy plasma HIV-1 RNA levels (log_10_ copies/mL) on persistent low-level viremia.

**Table 2:** Log-normal model parameter estimates for on-therapy plasma HIV-1 RNA levels as a function of baseline pre-therapy plasma HIV-1 RNA levels.

Parameter	Coefficient	P-value
**Constant**	-2.700	<0.001
**Pre-therapy plasma HIV-1 RNA (log**_**10**_ **copies/mL)**	0.772	<0.001
**Scale**	1.25	

#### Between-Patient Variability

Between-patient variability in cell concentrations in plasma has been introduced by exploiting the stable-state solution of the untreated (*ε* = 0), disease progression free (*f*_*p*_ = 1 and *f*_*s*_ = 1) system of ordinary differential equations. For untreated individuals, the contribution of the latently infected CD4^+^ T cell population to plasma HIV-1 RNA levels is negligible. Ignoring the presence of latently infected CD4^+^ T cells, two key parameters of the HIV-D model, the HIV-1 infection rate of CD4^+^ T cells ($k_{T_{1}}$) and the viral production rate (*p*_*V*_), can be written as a function of the stable-state plasma concentrations of target CD4^+^ T cells (${\overset{-}{T}}_{1}$) and HIV-1 virions ($\overset{-}{V}$), as

$$\begin{array}{lll} k_{T_{1}} & = & \frac{s_{T_{1}}-\delta_{T_{1}}{\bar{T}}_{1}}{\bar{V}{\bar{T}}_{1}}\\ p_{V} & = & \frac{\delta_{V}\delta_{T_{2}}}{k_{T_{1}}{\bar{T}}_{1}}. \end{array}$$ (4)

Both productively infected activated and latently infected resting CD4^+^ T cells represent only a residual fraction of the total CD4^+^ T cell population. As a result, the total CD4^+^ T cell plasma concentration (*T*) can be approximated by the target CD4^+^ T cell population (*T* ≅ *T*_1_). Between-patient variability in plasma cell concentrations over time can then be controlled by allowing for variability in model parameters $k_{T_{1}}$ and *p*_*V*_, substituting the desired viral set-point HIV-1 virion (*V*_*s*_) and total CD4^+^ T cell (*T*_*s*_) concentrations for $\overset{-}{V}$ and ${\overset{-}{T}}_{1}$ in the equations above.

#### Model Parameters

All base parameters relevant to the HIV-D model can be found in Table 3. Most parameters were initially taken from the literature [35, 39, 43]. Some were recalibrated to suit the purpose of modeling HIV-1 disease progression.

**Table 3:** Parameter values used in the basic HIV-D model.

Parameter	Description	Value	Source
$s_{T_{1}}$	Source rate of uninfected CD4+ T cells	10 cells/mm^3^/day	[35]
$k_{T_{1}}$	HIV-1 infection rate of CD4+ T cells	0.00036/virion/day^‡^^,^^¥^	[35]
$\delta_{T_{1}}$	Death rate of uninfected CD4+ T cells	0.01/day	[35]
***α***_***L***_	Percentage of latently infected CD4+ T cells	0.0015	[43]
$\delta_{T_{2}}$	Death rate of productively infected CD4+ T cells	0.7/day	[35]
***a***_***L***_	Activation rate of latently infected CD4+ T cells	0.01/day	[43]
***δ***_***L***_	Death rate of latently infected CD4+ T cells	0.004/day	[43]
***p***_***V***_	Viral production rate	70 virions/day/cell^‡^	[35]
***δ***_***V***_	Death rate of virions	23/day	[35]
***V***_***ref***_	Viral load reference level	100.000^¥^	[39]
***σ***	Scale parameter of Weibull CDF *Q*(*V*_*b*_)	5^¥^	[39]
***k***	Shape parameter of Weibull CDF *Q*(*V*_*b*_)	2^¥^	[39]
***p***	Viral production rate increase parameter	40^¥^	[39]
***ε***	Drug potency (% of new infections inhibited)	Variable*	
***V***_***Min***_	Minimal viral load (for persistent low viremia)	Variable^†^	

^‡^ See “between-patient variability”;

* See “PK/PD modeling”;

^†^ See “persistent low viremia”;

^¥^ Recalibrated

**Fig 3:**
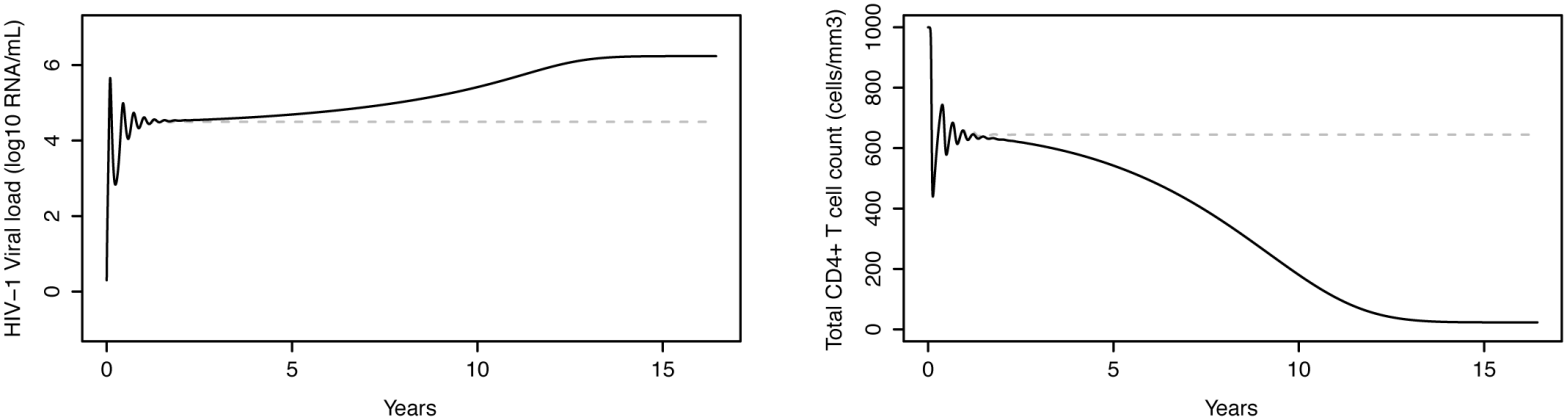
Evolution of plasma HIV-1 RNA levels and CD4+ T cell counts in an untreated subject. Left: Plasma HIV-1 RNA levels as a function of time, simulated by the HIV-D model accounting for disease progression. Right: corresponding CD4^+^ T cell counts. The model simulates the 3 typical stages of HIV-1 infection: acute infection, clinical latency and AIDS phase. Dashed grey lines represent the HIV-D model without disease progression, the stable-state solution of which is exploited to introduce between-patient variability in plasma cell concentrations.

Many different values can be found in the literature for the viral production rate (*p*_*V*_) and the HIV-1 infection rate of CD4^+^ T cells ($k_{T_{1}}$). For the base-case examples, presented throughout the paper, the viral production rate (*p*_*V*_) was fixed [35]. The HIV-1 infection rate ($k_{T_{1}}$) was calibrated, from within a realistic range of values [35], as for stable-state CD4^+^ T cells count and HIV-1 viral load to present suitable values (Fig 3). In the section on between-patient variability, it has been explained how these two parameters can be varied to introduce variability in stable-state CD4^+^ T cell counts and HIV-1 viral loads for different patients.

The remaining parameters that were subject to recalibration are related to the HIV-1 disease progression module. The conceptual model for disease progression was taken from a paper on co-infection with HIV-1 and tuberculosis, based on a different HIV-1 dynamics model [39]. Recalibration of the model parameters was necessary to result in a realistic CD4+ T cell decline and HIV-1 viral load increase over time (Fig 3 in the results section).

#### Disease Monitoring

Not all individuals in the simulated population are forced to perform regular HIV-1 testing. For those that do, HIV-1 testing is assumed to follow a Poisson process, with exponential times between consecutive tests. Upon being diagnosed, a fraction of infected individuals may choose not to seek treatment. For those who do seek treatment, regular medical visits are programmed in which CD4^+^ T cell counts and plasma HIV-1 RNA levels are monitored until a patient is considered eligible for treatment (e.g.: CD4^+^ T cell count below 500 or 350 cells/mm^3^ [9, 44]).

The model is completely flexible with respect to disease monitoring parameters, making it possible to investigate the effect on results of different hypotheses in terms of patients’ willingness to test, willingness to be treated, monitoring frequency and treatment eligibility.

### Primary Treatment Regimen Model

#### Adherence to Therapy

A quantitative relationship can be observed between the duration of the longer treatment interruption and the average adherence level [45], in which treatment interruptions of 30 or more days are not unusual for patients with an average adherence between 40% and 60%. Furthermore, there is a differential impact of adherence and treatment interruptions on the risk of virologic rebound (> 400 plasma HIV-1 RNA copies/mL) when comparing NNRTI- based ART to ritonavir-boosted (PI/r) based ART [45]. For NNRTI, due to their long terminal pharmacokinetic tail, at low-to-moderate adherence levels, sustained treatment interruptions pose a greater risk of virologic rebound than a comparable number of interspersed missed doses. On the other hand, PI/r average adherence rather than consecutive missed doses is found to be associated with virologic rebound. In raltegravir-based regimens longer treatment interruption and average adherence were both independently associated with virological failure [46]. Lastly, and most importantly, the quantitative relationship between the longer treatment interruption and the average adherence level seems to depend little on specific treatment regimens [45].

The correct modeling of the daily adherence process, in order to capture the delicate relationship between treatment interruptions and average adherence, is of utmost importance. Assuming adherence is a memoryless process may be misleading because the probability of a treatment interruption of 30 or more days, related to an average adherence of 50%, is almost non-existing. As such, in EPICE-HIV, adherence is modeled using a binary autoregressive model, assuming adherence on any given day is a random process, conditional on adherence on the previous day.

It depends on two key parameters: the probability of taking drugs on the current day, given drugs were skipped the previous day (*p*_10_) or not (*p*_11_). For each individual in the simulated population, an expected average adherence level (*α*_*Adh*_) is sampled from a pre-defined empirical or theoretical population-level distribution. Given this adherence level, the parameters *p*_10_ and *p*_11_ are then determined randomly for each individual as

$$\begin{array}{lll} p_{10} & \sim U\left(0.10;1.00\right) & \text{if }\alpha_{Adh}\geq 0.80,\\ p_{10} & \sim U\left(0;\text{min}\left[0.25;\frac{\;\alpha_{Adh}}{1-\;\alpha_{Adh}}\right]\right) & \text{if }\alpha_{Adh}\geq 0.80,\\ p_{11} & =1+p_{10}-\frac{p_{10}}{\alpha_{Adh}}. & \end{array}$$ (5)

Here, *p*_10_ is sampled from one of two possible continuous uniform distributions, conditional on the expected average adherence level being inferior or superior to 80%. The uniform distributions were calibrated as to result in a qualitatively representative simulated relationship between the longer treatment interruption and average adherence [45]. No formal calibration techniques were used. *p*_11_ Is determined as a function of *p*_10_ and *α*_*Adh*_ to ensure that the adherence level of each individual converges over time to the pre-specified average adherence level *α*_*Adh*_.

#### PK/PD Modeling

Normalized drug concentrations *C*_*n*_(*t*) = *C*(*t*)/*IC*_50_ (with *C*(*t*) the drug concentration and *IC*_50_ the 50% inhibitory concentration against wild-type virus) are modeled as a function of time as in [47] using the impulsive differential equation:

$$\begin{array}{lll} \frac{dC_{n}}{dt} & =-\;w_{C}C_{n}\;, & t\neq \tau_{i},\;\left(\text{no dose taken}\right),\\ C_{n}\left(t^{+}\right) & =C_{n}\left(t^{-}\right)+C_{n,sd}, & t=\tau_{i},\;\left(\text{dose taken}\right). \end{array}$$ (6)

Normalized concentration *C*_*n*_(*t*) is assumed to increase instantaneously by a single amount (*C*_*n*,*sd*_) every time-point *τ*_*i*_ (for *i* = 1,2, …) a dose is taken, while in between doses *C*_*n*_(*t*) decays exponentially with a certain rate *w*_*C*_. For most commercially available antiretroviral drugs, values are available for their 50% inhibitory concentration (*IC*_50_), plasma half-life (*t*_1/2_), required dosing interval (*δτ = τ*_i+1_−*τ*_*i*_) and related maximum concentration (*C*_*max*_). These values allow for *w*_*C*_ and *C*_*n*,*sd*_ to be determined as follows:

$$\begin{array}{lll} w_{C} & = & \frac{\text{ln}\left(2\right)}{t_{1/2}},\\ C_{n,sd} & = & \frac{C_{max}}{IC_{50}}\left(1-e^{-\;w_{C}\delta_{\tau }}\right). \end{array}$$ (7)

The drug-potency *ε*_*wt*_ against wild-type HIV-1 virus for a given drug is modeled as a function of its normalized concentration using a Hill type dose-response curve [48]:

$$\begin{array}{rrr} 1-\varepsilon_{wt} & = & \frac{1}{C_{n}^{m}+1} \end{array},$$ (8)

representing the fraction of new infections unaffected by the drug. A logarithmic measure of inhibition *F*_*wt*_ can be defined from this equation, as

$$\begin{array}{rrrrr} F_{wt} & = & \text{log}\left(\frac{\varepsilon_{wt}}{1-\varepsilon_{wt}}\right) & = & m\times \text{log}\left(C_{n}\right) \end{array}.$$ (9)

The slope parameter *m* of the linearized dose-response curve has greatly been ignored when describing the antiviral activity of therapy in HIV-1 infection, but is of great importance to determine inhibition at clinical drug concentrations (*C*_*n*_ ≫ 1) [49].

For combination therapy, drug potency interaction effects can readily be modeled, starting from the individual normalized drug concentrations, through Loewe additivity or Bliss independence [49]. Loewe additivity assumes drugs have similar mechanisms or compete for the same binding site. For a three-drug version of the Loewe additivity model, drug potency *ε*_*wt*,*L*_ can be described by the equation:

$$\begin{array}{rrr} 1 & = & C_{1,n}{\left(\frac{1-\varepsilon_{wt,L}}{\varepsilon_{wt,L}}\right)}^{\frac{1}{m_{1}}}+C_{2,n}{\left(\frac{1-\varepsilon_{wt,L}}{\varepsilon_{wt,L}}\right)}^{\frac{1}{m_{2}}}+C_{3,n}{\left(\frac{1-\varepsilon_{wt,L}}{\varepsilon_{wt,L}}\right)}^{\frac{1}{m_{3}}}. \end{array}$$ (10)

Bliss independence assumes independent action between drugs, such that the combined effect *ε*_*wt*,*B*_ of triple combination therapy can be written as the product of the fractions unaffected by each individual drug:

$$\begin{array}{rrr} 1-\varepsilon_{wt,B} & = & \frac{1}{C_{1,n}^{m_{1}}+1}\times \frac{1}{C_{2,n}^{m_{2}}+1}\times \frac{1}{C_{3,n}^{m_{3}}+1}. \end{array}$$ (11)

It predicts higher inhibition than Loewe additivity, especially at clinical drug concentrations. Inhibition less than the Loewe prediction represents antagonism, whereas a combined effect significantly greater than the Bliss prediction represents synergy.

The inhibitory effect of drug combinations are characterized on a spectrum defined by two states, independent inhibition (Bliss independence) and competitive binding (Loewe additivity), with synergistic and antagonistic interactions at either extreme [49], as

$$\begin{array}{rrr} F_{wt,DI} & = & F_{wt,L}+DI\times \left(F_{wt,B}-F_{wt,L}\right). \end{array}$$ (12)

where *F*_*wt*,*DI*,_ *F*_*wt*,*L*_ and *F*_*wt*,*B*_ follow the definition of the previously introduced logarithmic measure of inhibition. Here, *DI* represents an index that quantifies the degree of independence, with *DI* = 0 corresponding to Loewe additivity and *DI* = 1 to Bliss independence.

The inhibitory potential at clinical drug concentrations for pairwise and triple combinations of 19 commonly used antiretrovirals were analyzed, allowing for quantitative comparison of antiviral activity of different drug regimens at expected plasma concentrations [49]. The results of this analysis in terms of *DI* index estimates can be used to determine the combined inhibition effect of different drug regimens in the spectrum defined by Bliss independence and Loewe additivity.

A summary of key PK/PD modeling quantities can be found in Table 4.

**Table 4:** Key PK/PD modeling quantities.

Quantity	Description
***C*(*t*)**	Drug concentration, as a function of time
***IC***_**50**_	50% Inhibitory concentration (against wild-type virus)
***C***_***n***_**(*t*)**	Normalized drug concentration, as a function of time
***C***_***n*,*sd***_	Instantaneous single dose increase in normalized drug concentration
***w***_***C***_	Exponential decay rate of normalized drug concentration
***ε***_***wt***_	Drug potency (against wild-type virus), *C*_*n*_ dependent
***F***_***wt***_	Logarithmic function of inhibition (against wild-type), *C*_*n*_ dependent
***m***	Slope parameter of logarithmic function of inhibition (*F*_*wt*_)
***DI***	Degree of independence
	Loewe additivity *DI* = 0
	Bliss independence *DI* = 1

#### Disease Monitoring

Viral suppression is checked for the first time at 1–2 months for a patient starting a new ART and every 4–6 months thereafter, following international treatment guidelines [9, 44]. If HIV-1 viral load is suppressed (<50 plasma RNA copies/mL), regular medical follow-up visits are programmed for plasma HIV-1 RNA and CD4^+^ T cell count monitoring. If on any of these medical visits plasma HIV-1 RNA is no longer suppressed, a confirmation visit is scheduled (e.g. one month after [9]). If plasma HIV-1 RNA continues non-suppressed, the patient is defined as having a virologic failure and moves to a subsequent treatment line. If plasma HIV-1 RNA is suppressed again, the patient maintains its current treatment regimen and returns to regular medical follow-up visits. Any time during the treatment, a patient can switch a regimen due to adverse events or other non-virologic reasons. Time to event distributions are considered for these regimen switches.

#### Costs

Daily costs for each of the components in the modeled ART are quantified independently and accumulated continuously (dependent on adherence) for ART cost identification purposes. For non-ART costs (e.g. non-ART medication, examinations and laboratory tests) the model follows the same rules as for secondary treatment regimens discussed below. The same holds for the simulation of hospitalizations and corresponding costs.

### Secondary Treatment Regimens Model

#### Suppressive Therapy

Modeling of suppressive therapy for regimens of secondary interest is based on an adaptation of a DES-based cost-effectiveness model [34]. Conditional on a number of patient-specific characteristics (age, gender, regimen characteristics, resistance level, etc.) the time to the occurrence of 5 clinical events is estimated:

- Viral suppression (plasma HIV-1 RNA <50 copies/mL)
- Regimen switch due to virologic failure
- Regimen switch not due to virologic failure (e.g.: toxicity)
- Resistance development
- Hospitalization

This section provides a concise description of the modeling steps. Further details can be found in the original publication [34].

Weibull distributions are considered for the estimation of event times for each of the clinical events. Upon the occurrence of any event (except hospitalization), time to occurrence of all events is updated using a conditional distribution approach. Virologic failure is defined as a confirmed plasma HIV-1 RNA level >50 copies/mL after initial viral suppression or unreached viral suppression 6 months after therapy initiation.

With respect to time to viral suppression, the plasma HIV-1 log_10_ RNA level is assumed to decline linearly from the current value until reaching the value 0.5 (corresponding approximately to 3 plasma HIV-1 RNA copies/mL) at the estimated time, and is kept constant until virologic failure. Plasma HIV-1 log_10_ RNA rebound levels for virologic failure are sampled from a uniform distribution, with parameters calibrated on observational data [34]. Meanwhile, the evolution of CD4^+^ T cell counts is governed automatically by the basic HIV-D model. Patients may switch regimens for reasons other than virologic failure an unlimited number of times within each treatment line. When a regimen switch due to virologic failure occurs, patients move to a subsequent treatment line. Upon regimen switch due to virologic failure on third-line treatment, patients loop back to this treatment line as many times as necessary until resistance reaches the highest class, regardless of the number of virologic failures. Once the highest resistance class is reached upon line switch, individuals start non-suppressive therapy.

Resistance levels are grouped into four classes, according to the inverted Genotypic Sensitivity Score [34, 50] and it is assumed that patients can move only to an adjacent, higher resistance class because resistance is archived in cellular DNA. Upon reaching the time to (new) resistance development, resistance levels are sampled uniformly from within each class. Adherence evolves over time based on a generalized linear model. Regimen characteristics are updated on occurrence of line and regimen switches.

#### Non-Suppressive Therapy

Upon reaching non-suppressive therapy, the plasma HIV-1 log_10_ RNA level is set to a value sampled from a normal distribution with parameters calibrated on observational data [31]c. After this, modeling of CD4^+^ T cell counts and plasma HIV-1 RNA levels is returned back to the HIV-D model. As a result of the increasing total viral burden, plasma HIV-1 RNA levels keep increasing whereas CD4^+^ T cell counts start dropping to AIDS-defining levels, in turn increasing mortality risk.

#### Costs

The model considers monthly ART and non-ART costs, both based on generalized linear models, which are updated upon regimen switch and changes in resistance level. Monthly non-ART costs include in addition, among others, physician appointments, exams and laboratory tests [34].

### Mortality and Quality of Life

Before, it was argued how, during the acute stage of the infection CD4^+^ T cell counts initially drop and then increase rapidly, however never returning to pre-infection levels. If untreated, the subsequent clinical latency stage of the infection is characterized by a slow depletion of CD4^+^ T cells until reaching AIDS-defining levels, typically about 8–10 years after seroconversion. If treated, CD4^+^ T cell counts grow slowly, but again usually do not reach pre-infection levels.

Studies show that mortality is CD4^+^ T cell count dependent and, even after ART initiation, remains higher in HIV-1 infected individuals than in the general population [51]. For HIV-1 infected individuals with CD4^+^ T cell counts below 200 cells/mm^3^, a 30-fold increase in (age and gender standardized) mortality rates has been estimated, whereas for patients with CD4^+^ T cell counts above 500 cells/mm^3^, this is reduced to a 2.5-fold increase [51].

At model initiation, the life expectancy of each simulated individual is sampled from mortality tables of the general population. Instantaneous HIV-1 infection related excess mortality as compared to the general population is implemented using CD4^+^ T cell count-dependent standardized mortality ratios (SMR, Table 5) [51]. The model is completely flexible with respect to SMR, easily adaptable as more data on the true impact of life expectancy is acquired. In order to keep the total population-size constant, each time an individual dies, a new, uninfected individual is generated, aged 13-years old (considered the minimum age for onset of sexual availability) [32].

**Table 5:** Standardized mortality ratios for excess mortality due to HIV-1 infection.

Parameter	CD4+ T cell count	Value
**Standardized Mortality Ratio**	≥500 cells/mm^3^	2.5
	500 cells/mm^3^–350 cells/mm^3^	3.5
	350 cells/mm^3^–200 cells/mm^3^	5.6
	<200 cells/mm^3^	30.3

Given the variable nature of CD4^+^ T cell counts (disease progression and ART dependent) in the model, it is easily understood that CD4^+^ T cell counts are one of the main drivers of mortality in EPICE-HIV.

Similarly, CD4^+^ T cell-dependent quality of life (QoL) utilities are considered for cost-effectiveness modeling in terms of quality adjusted life years (QALY). Two sets of utilities are considered: one for plasma HIV-1 RNA suppressed individuals, and another for unsuppressed individuals (Table 6) [49].

**Table 6:** Quality of life utilities for cost-effectiveness modeling.

Parameter	CD4+ T cell count	Suppressed	Unsuppressed
**QoL Utility**	≥500 cells/mm^3^	0.954	0.938
	500 cells/mm^3^–350 cells/mm^3^	0.934	0.931
	350 cells/mm^3^–200 cells/mm^3^	0.929	0.932
	200 cells/mm^3^–50 cells/mm^3^	0.863	0.849
	<50 cells/mm^3^	0.781	0.781

### External Model Validation

Due to the complexity and comprehensiveness of the EPICE-HIV model as a whole, components that have been previously validated or described in the literature are not presented nor discussed. Though constituting a limitation to the current paper, validation within EPICE-HIV of these model components will be addressed in future work. The secondary treatment regimen part of the within-host model and the transmission module have previously been discussed in detail within comparable contexts [32, 34]. As such, the present analysis focuses on the HIV-D model and the implementation of the primary treatment regimen in the within-host model.

To strengthen the external validity of the HIV-D model, a simulation study was performed to allow comparisons to the data from the RPV/FTC/TDF arm of the STaR study [33]. The STaR study was a phase 3b, randomized, multicenter, international, open-label, 96-week clinical trial comparing the safety and efficacy of two single-tablet regimens, RPV/FTC/TDF and EFV/FTC/TDF, in 786 ART-naive, HIV-1 infected patients. Full validation against STaR study results, also including once-daily treatment with coformulated EFV/FTC/TDF will be discussed in an upcoming paper. In future work, the validation of the model against data from observational studies will also be considered.

The simulation consisted of 100,000 hypothetical infected individuals, initiating first-line ART with once-daily, coformulated RPV/FTC/TDF, using the same set of conditions in terms of baseline characteristics, clinical management and efficacy criteria of the STaR study [5]. We tested the hypothesis if the HIV-D model component of EPICE-HIV would be able to accurately produce 96-week percentages of virologically suppressed patients (as determined by the FDA Snapshot algorithm), qualitatively and quantitatively comparable to the percentages observed in the STaR study [33], and what effect alternative adherence distributions have on the results.

## Results and Discussion

### HIV-1 Dynamics Model

#### Disease Progression

As an independent module, in the absence of drug effect, the HIV-D model simulates CD4^+^ T cell counts and plasma HIV-1 RNA levels as a function of time for HIV-1 infected individuals that pass through the 3 typical stages of HIV-1 infection: acute infection, clinical latency and AIDS phase (Fig 3) [52].

The HIV-D model is capable of reproducing not only the acute stage of HIV-1 infection, but also the slow depletion of CD4^+^ T cells and increase in viral load typical of the clinical latency stage. In the simulated example, after about 8 years from seroconversion, the CD4^+^ T cell count steadily declines, reaching the value of 200 cells/mm^3^ at 9.7 years after infection.

#### Drug Potency

**Fig 4:**
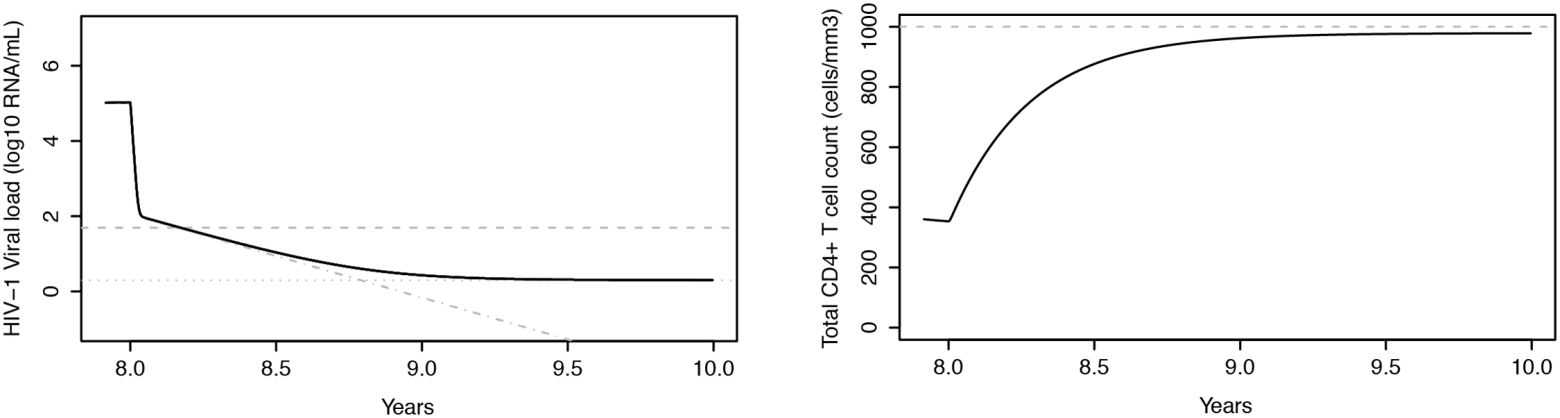
Evolution of plasma HIV-1 RNA levels and CD4+ T cell counts in a treated subject. Left: plasma HIV-1 RNA levels as a function of time, simulated by the HIV-D model with drug effect, when persistent (black line) and non-persistent (grey dashed-dotted line) low level viremia are considered. The horizontal grey dashed line corresponds to the common viral load detection threshold of 50 plasma HIV-1 RNA copies/mL, whereas the horizontal grey dotted line corresponds to a theoretically imputed persistent low level viremia of 2 plasma HIV-1 RNA copies/mL (considered for this particular example). Right: corresponding CD4+ T cell counts. Simulated treatment with hypothetical continuous drug potency ε = 100% starting at 8 years after seroconversion.

In Fig 4 the evolution of the CD4^+^ T cell count and plasma HIV-1 RNA levels is presented as a function of time for a patient starting ART 8 years after seroconversion with hypothetical continuous drug potency *ε* = 100%,.

After treatment initiation, the CD4^+^ T cell count grows slowly, but does not reach the pre-infection level of 1000 cells/mm^3^. This is a consequence of the inclusion of disease progression in the model, as previously mentioned. The decline in plasma viral load follows the three phases described before. A sharp drop in viral load occurs within the first two weeks of ART, determined mainly by the decay rate of productively infected activated CD4^+^ T cells (*T*_2_). Afterwards the viral load keeps declining, but at a much slower rate (decay rate of latently infected CD4^+^ T cells). The threshold of 50 plasma HIV-1 RNA copies/mL is reached after 2 months, but can be adjusted depending on the family of antiretroviral drugs used. Viral persistence at a level of about 2 plasma HIV-1 RNA copies/mL (considered for this particular example) is set in approximately 1 to 1.5 years after treatment initiation.

The grey dashed-dotted line in the left-hand side graph of Fig 4 represents the evolution of the viral load when the persistent low level viremia adjustment to the HIV-D model is ignored. The second-phase decline then takes the viral load to unreliable levels, demonstrating the need for explicit persistent low viremia modeling.

### Primary Treatment Regimen

#### Adherence to Therapy

**Fig 5:**
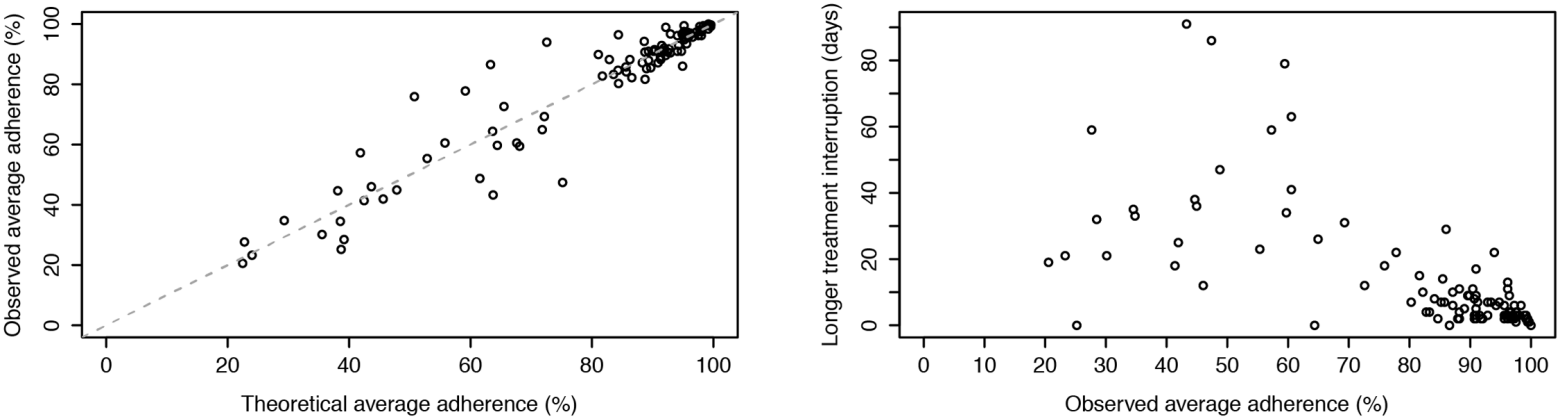
Simulated average adherence and treatment interruption duration. Left: Correlation between the theoretical average adherence and simulated observed average adherence, obtained with the first-order binary autoregressive adherence model, for a random sample of 100 hypothetical individuals followed for a 1-year time-period. Theoretical average adherence distribution: 30% with adherence below 80%; 20% with adherence between 80% and 90%; 50% with adherence above 90%. Right: Relationship between simulated observed average adherence and corresponding longer treatment interruption, for the same random sample.

Using the binary first-order autoregressive adherence model, Fig 5 shows there is not only a strong correlation between the theoretical desired average adherence and the final observed simulated average adherence, but there is also a relationship between the longer treatment interruption and the observed average adherence, quantitatively and qualitatively comparable to the one found in Parienti et al. (2010) [45]. The simulation is based on a random sample of 100 hypothetical individuals followed for over a 1-year time period, with theoretical average adherence distribution: 30% with adherence below 80%; 20% with adherence between 80% and 90%; and 50% with adherence above 90% [45].

#### PK/PD Modeling

The results presented below focus on a once-daily, triple combination therapy with coformulated rilpivirine 25mg (RPV), emtricitabine 200mg (FTC) and tenofovir DF 245mg (TDF) and are based on PK parameters from Table 7.

**Table 7:** PK/PD modeling parameters for RPV, FTC and TDF.

Parameter	RPV [48, 53]	FTC [49]	TDF [48, 49]
***C***_***max***_	0.378 μMol	6.8 μMol	1.06 μMol
***IC***_**50**_	0.0039 μMol	0.0112 μMol	0.013 μMol
***t***_**1/2**_	45 h	39 h^‡^	60 h^‡^
***m***	1.92	0.92	0.42

^‡^ Intracellular

**Fig 6:**
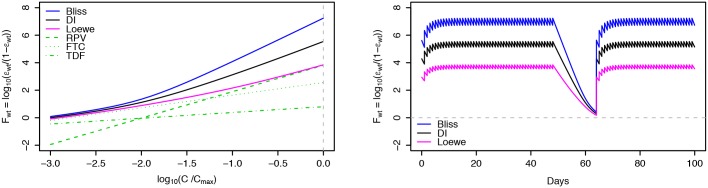
Drug inhibition as a function of drug concentration and time. Left: Logarithmic measure of inhibition (F_wt_) as a function of drug concentration (percentage of maximum concentration, log_10_-scale), for drugs RPV, FTC and TDF alone (green lines) and predictions of the combined effect of RPV+FTC+TDF by the Bliss, Loewe and DI model. For the DI model, a hypothetical index 0.5 of degree of independence was assumed. The vertical grey dashed line represents the inhibition potential at maximum concentration for all three drugs. Right: Logarithmic measure of inhibition (F_wt_) as a function of days, based on the Bliss, Loewe and DI model combined effect predictions. Simulations are representative of triple combination ART initiation with RPV+FTC+TDF, for a patient 100% adherent to therapy, except for a treatment interruption of 15 days, starting at day 50. After 15 days, a level of only 50% inhibition, represented by the horizontal grey dashed line, starts being approached.

The logarithmic measure of inhibition (*F*_*wt*_), was calculated as a function of drug concentration (log-scale percentage of maximum concentration, Fig 6) for RPV, FTC and TDF. At clinical concentrations, immediately to the left of the vertical grey dashed line, neither of the individual drugs attains a value larger than 5, suggested as a minimum threshold for successful HAART [46]. Predictions are also presented for the combined inhibition effect of RPV+FTC+TDF by the Bliss independence, Loewe additivity and *DI* model. For the *DI* model, a hypothetical index 0.5 of degree of independence was assumed. Intermediate inhibition can be defended for NNRTI-NRTI interactions [49]. For the Bliss independence and *DI* model predictions, inhibition values are above 5. For the Loewe additivity model predictions, inhibition values are mainly driven by the NNRTI component (RPV) of the ART regimen.

On the right-hand side of Fig 6, corresponding logarithmic measures of inhibition are presented as a function of days, for an EPICE-HIV simulated patient, 100% adherent to therapy, except for a treatment interruption of 15 days, starting at day 50. During this treatment interruption, inhibition is seen to drop fast, ultimately approaching the level of 50% inhibition (horizontal grey dashed line), suggesting an increase in viral replication. At the end of the treatment interruption, the decline in inhibition effect seems to slow down slightly, owing mainly to the lower slope parameters *m* of the NRTI. As can be seen further, after the reintroduction of therapy, it takes about another 15 days for the inhibition effect to converge back to its periodic orbit, prolonging the impact of the treatment interruption on inhibition.

Jilek et al. (2012) [49] further investigated the correlation between clinical outcomes of RCT and IIP values of different drugs and drug combinations, suggesting that for ART regimens with low IIP, the percentage of patients with plasma HIV-1 RNA levels below 50 copies/mL at 48 weeks is correlated to the estimated IIP of the considered regimen. For ART regimens with high IIP, on the other hand, it is argued that little correlation with outcome can be detected, suggesting that at high IIP levels, viral replication is mostly halted and outcomes depend mainly on adherence to therapy.

#### Combining the Adherence, PK/PD and HIV-D Models

**Fig 7:**
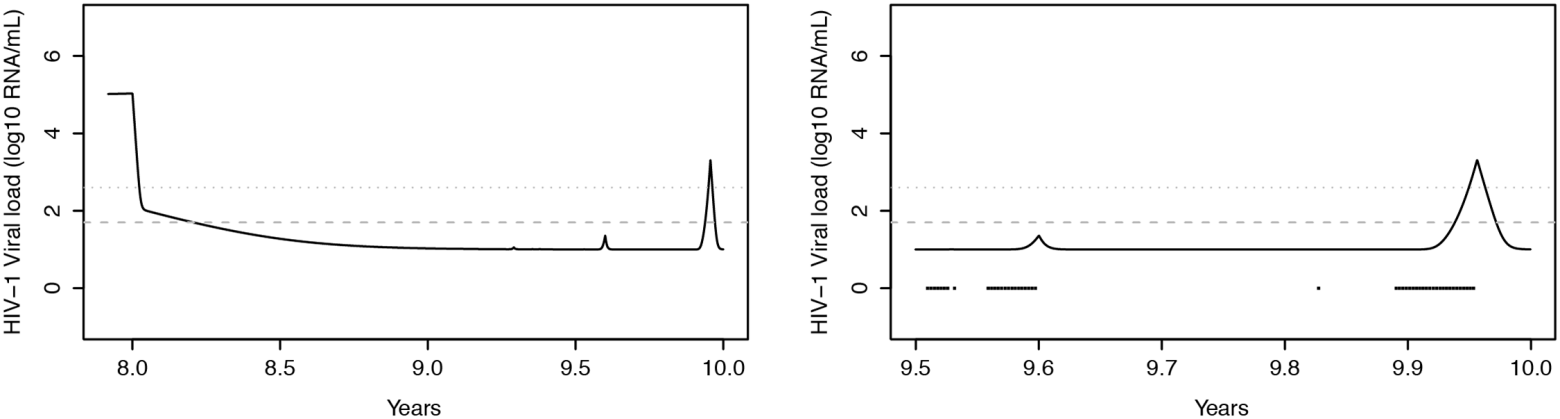
Treatment interruptions and viral response. Left: Plasma HIV-1 RNA levels as a function of time, simulated by the HIV-D model with drug effect modeled by the primary treatment regimen module of EPICE-HIV. The treatment considered is RPV/FTC/TDF, starting at 8 years after seroconversion with a theoretically imputed persistent low viremia level of 10 plasma HIV-1 RNA copies/mL. For comparability with Parienti et al. [45, 54] during the first year of therapy, 100% adherence was assumed, allowing for plasma HIV-1 RNA levels to be controlled (below 50 copies/mL). After that, adherence to therapy is driven by the first-order binary autoregressive adherence model. In the second year of therapy, the patient demonstrated 76% of adherence. Right: Close-up of the last half year of therapy, demonstrating two large treatment interruptions, represented by black squares: one of 15 days (around 9.6 years) and another of 24 days (around 9.9 years). The horizontal grey dashed and dotted lines correspond to viral load detection thresholds of 50 and 400 plasma HIV-1 RNA copies/mL, respectively. Though the 15 day interruption leads to drug concentrations that allow for viral replication, it is not long enough for plasma HIV-1 RNA levels to rise above the detection thresholds. The 24 day interruption leads to plasma HIV-1 RNA levels above 400 copies/mL. Several days after reintroduction of treatment, levels remain detectable by the 50 copies/mL threshold.

To demonstrate the interaction of the first-order binary autoregressive adherence model with the PK/PD model and HIV-D model within EPICE-HIV, a simulated patient was considered, starting coformulated RPV/FTC/TDF 8 years after seroconversion and followed-up for 2 years. For the purpose of this particular simulation, a theoretical persistent low level viremia of 10 plasma HIV-1 RNA copies/mL was considered and an index of degree of independence *DI* of 0.5 was assumed. During the first year of therapy the patient was simulated to be 100% adherent, allowing for RPV/FTC/TDF to be successful in controlling plasma HIV-1 RNA levels below 50 copies/mL (Fig 7).

After the first year of therapy, adherence was allowed to be driven by the first-order binary autoregressive adherence model. During the second year of therapy, the patient demonstrated an average adherence of 76%, leading to two major continuous treatment interruptions (Fig 7, black squares). The first occurred just before 9.6 years, with a duration of 15 days. The second occurred around 9.9 years, with a duration of 24 days. The 15 day treatment interruption leads to drug concentrations that allow for viral replication, showing a small increase in plasma HIV-1 RNA levels (Figs 6 and 7). The treatment interruption is not long enough though, to warrant detection at a 50 copies/mL threshold. The 24 day interruption, on the other hand, is long enough for plasma HIV-1 RNA levels to rise not only above 50 copies/mL, but also above 400 copies/mL. Furthermore, several days after the reintroduction of treatment, viral load levels remain detectable by the 50 copies/mL threshold, owing to the time it takes for viral inhibition to converge back to its desired levels.

A previous logistic model has estimated a ≥ 90% probability of virologic rebound (plasma HIV-1 RNA ≥ 400 copies/mL) associated to treatment interruptions of 24 days for NNRTI-based ART with comparable follow-up, making results observed at the end of the simulated two-year treatment period in Fig 7 more than plausible [54]. For treatment interruptions of 15 days, the estimated probability of virologic rebound was 50%.

Further simulations have shown that if persistent levels of viremia are set higher (e.g.: closer to 40 copies/mL), a similar adherence pattern leads to plasma HIV-1 RNA levels above 50 copies/mL at the end of a 15 day treatment interruption. This highlights the importance of correct modeling of low level viremia. Even with lower levels of persistent viremia, if several small, closely interspaced treatment interruptions would precede the longer 15 day treatment interruption, plasma HIV-1 RNA levels would likely be low, but uncontrolled before the start of the 15 day interruption, augmenting the probability of virologic rebound at the end of the interruption. This suggests that not only the duration of treatment interruptions, but also their inter-location is important. Both situations occur in a natural way within EPICE-HIV, making virologic rebound at 400 copies/mL plausible for a 15 day treatment interruption, under the right circumstances.

### Using the STaR Trial for External Model Validation

The main results comparing the simulated 96-week percentages of virologically suppressed EPICE-HIV simulated patients initiating first-line ART with RPV/FTC/TDF to the corresponding percentages observed in the STaR study [33], can be found in Table 8.

**Table 8:** Week 96 snapshot analysis for the RPV/FTC/TDF arm of the STaR study [33] and the simulated population in the EPICE-HIV model: detailed results.

	STaR Trial Data		EPICE-HIV Simulation	
	FAS	MAS	Base Case	Alternative
**Virologic Success at Week 96**	**77.9%**	**79.3%**	**82.7%**	**78.6%**
HIV-1 RNA < 50 copies/mL	77.9%	79.3%	82.7%	78.6%
**Virologic Failure at Week 96**	**9.4%**	**9.6%**	**4.0%**	**8.5%**
HIV-1 RNA ≥ 50 copies/mL	1.5%	1.6%	0.3%	0.7%
Discontinued study drug due to lack of efficacy	4.1%	4.1%	1.0%	5.1%
Discontinued study drug due to other reasons and last available HIV-1 RNA ≥ 50 copies/mL	3.8%	3.9%	2.7%	2.7%
**No Virologic Data in Week 96 Window**	**12.7%**	**11.1%**	**13.3%**	**12.9%**
Discontinued study drug due to AE or death	3.0%	3.1%	3.3%	3.3%
Discontinued study drug due to other reasons and last available HIV-1 RNA < 50 copies/mL	7.9%	8.0%	10.0%	9.7%
Missing data during window but on study drug	1.8%	0.0%	0.0%	0.0%

**FAS**: Full analysis set; **MAS**: Modified analysis set (excludes patients on treatment, but with missing data during 96 week window; **Base case**: Simulations performed with the adherence distributions observed in the STaR study; **Alternative**: Simulations performed with alternative hypothetical adherence distributions, adjusting for possible bias in self-reported adherence.

STaR trial data comprises the full analysis set (FAS) and a modified analysis set (MAS), excluding patients with missing data at 96 weeks. The FAS population consists of the 394 patients randomized to and treated with RPV/FTC/TDF. Because our model provides exhaustive virologic information (no missing data) MAS results are also considered for comparability reasons. EPICE-HIV simulations are based on the adherence distribution from the STaR study (‘Base Case’) and an alternative adherence distribution (‘Alternative’, details below) to account for potential bias arising from self-reported adherence.

Comparing the EPICE-HIV estimates in the base case with the MAS results of the STaR study, most of the observed differences are a direct consequence of a slightly lower rate of virologic failure in the simulated data. At week 96, a smaller percentage of simulated cases had viral loads ≥50 copies/mL than in the STaR study (0.3% vs 1.6%). Similarly, discontinuations due to lack of efficacy showed a smaller percentage in the simulated population (1.0%) than in the STaR population (4.1%). Furthermore, also a smaller percentage of patients discontinuing due to other reasons had uncontrolled viral loads in the simulated data (2.7%) as compared to the STaR study (3.9%).

Overall, a virologic success rate of 82.7%, a virologic failure rate of 4.0% and a rate of absence of virologic data of 13.3% was simulated, compared to the rates of 79.3%, 9.6% and 11.1% in the MAS STaR trial data, respectively. The largest difference observed is with respect to the rate of virologic failure and is the only one reaching statistical significance (risk difference: -5.5%; 95%CI [-8.5%;-2.6%]) (Table 9).

**Table 9:** Week 96 snapshot analysis for the RPV/FTC/TDF arm of the STaR study [33] and the simulated population in the EPICE-HIV model: risk differences between simulated and observed results.

	STaR Trial Data	EPICE-HIV Simulation	
	MAS	Base Case	Alternative
**Virologic Success at Week 96**	**79.3%**	**82.7%**	**78.6%**
Risk difference—% (95% CI)		3.3% [-0.7%;7.4%]	-0.7% [-4.8%;3.3%]
**Virologic Failure at Week 96**	**9.6%**	**4.0%**	**8.5%**
Risk difference—% (95% CI)		-5.5% [-8.5%;-2.6%]	-1.1% [-4.0%;1.9%]
**No Virologic Data in Week 96 Window**	**11.1%**	**13.3%**	**12.9%**
Risk difference—% (95% CI)		2.2% [-1.0%;5.4%]	1.8% [-1.3%;5.0%]

**MAS**: Modified analysis set (excludes patients on treatment, but with missing data during 96 week window; **Base case**: Simulations performed with the adherence distributions observed in the STaR study; **Alternative**: Simulations performed with alternative hypothetical adherence distributions, adjusting for possible bias in self-reported adherence.

These observed differences might be related to multiple factors, an important one of which may be adherence. Adherence in the STaR study was determined through the Medication Adherence Self Report Inventory (MASRI) questionnaire, completed by study subjects. Studies on the relationship between self-reported adherence, electronically monitored adherence and virologic success [17, 18, 55] have suggested that self-reported adherence is generally higher than electronically monitored adherence and the likelihood of achieving virologic success is greater if electronically monitored adherence is high than if self-reported adherence is high. Differences of over 20% in average adherence when comparing self-reported to electronically monitored adherence can be found in the literature.

Though in a clinical trial setting, with a population less representative of the general population, this effect might be attenuated, it should not be ignored all together when simulating. Adherence in the STaR study was generally very high (average 96.8%) even after 96 weeks of treatment, with few patients demonstrating adherence levels below 80%. To test the hypothesis of the effect of the adherence distribution on the EPICE-HIV results, a second scenario was set up, considering an alternative distribution with an average 5% decrease in adherence as compared to the adherence data observed in the STaR study (Table 8).

The results suggest a higher rate of virologic failure as compared to the base case scenario, relating better to the results observed in the MAS population (Table 9). Individual differences can be interpreted as an effect of timing in treatment interruptions. Overall, 5.7% of the STaR population (MAS) suffered treatment failure (discontinuing study drug due to lack of efficacy, or ≥50 plasma HIV-1 RNA copies/mL) at week 96. For the simulated population, the alternative scenario would result in an estimate of 5.8%, closer to observed values than the 1.3% estimated in the base case.

In conclusion, using a fine-tuned multi-paradigm micro-simulation model, we were able to approximate the 96 week outcomes of subjects treated with coformulated RPV/FTC/TDF in the randomized STaR study.

### Practical Implications

With EPICE-HIV, we have argued and demonstrated that adherence to ART is an important driver of clinical and therapeutic outcomes in HIV-1 infected individuals. For optimal outcomes, high levels of adherence are required. Suboptimal adherence levels can have serious clinical, economic and epidemiologic implications.

Systematic lack of adherence to ART may lead to an increase in viral replication, especially–but not only–for therapies with short PK half-lives. On the one hand, increased viral replication is associated with reduced immunological response of patients, impacting the economic burden of the disease, quality of life and mortality. On the other hand, it also increases the risk of disease transmission, posing a clear public health issue. Consequently, from a health policy perspective, EPICE-HIV may be valuable in assessing and identifying optimal HIV-1 health care strategies—particularly those related to improving adherence—in a very comprehensive way.

EPICE-HIV further demonstrates that even small changes to adherence can affect clinical and economic outcomes, not only on the short-term, but also on the long-term, potentially changing the relative value of different therapy options. Given the difference in adherence typically observed between clinical practice and clinical trials, from a health technology assessment and cost-effectiveness perspective, EPICE-HIV may aid in defining the real-world clinical and economic value of different ART regimens for decision makers.

In the light of this, validation of EPICE-HIV against observational study data, which are more representative than randomized controlled trial data for the analysis of real-world clinical settings, should further strengthen the external validation of the model. This limitation to the current paper will be addressed in future work.
